# Fungal Carbon: A Cost‐Effective Tunable Network Template for Creating Supercapacitors

**DOI:** 10.1002/gch2.202300315

**Published:** 2024-03-18

**Authors:** Mitchell P. Jones, Qixiang Jiang, Andreas Mautner, Aida Naghilou, Alexander Prado‐Roller, Marion Wolff, Thomas Koch, Vasiliki‐Maria Archodoulaki, Alexander Bismarck

**Affiliations:** ^1^ Institute of Materials Science and Technology Faculty of Mechanical and Industrial Engineering TU Wien Gumpendorferstrasse 7, Objekt 8 Vienna 1060 Austria; ^2^ Polymer & Composite Engineering (PaCE) Group Institute of Materials Chemistry and Research Faculty of Chemistry University of Vienna Währinger Straße 42 Vienna 1090 Austria; ^3^ Institute for Environmental Biotechnology Department IFA University of Natural Resources and Life Sciences Vienna Konrad‐Lorenz‐Straße 20 Tulln an der Donau 3430 Austria; ^4^ Department of Plastic Reconstructive and Aesthetic Surgery Medical University of Vienna Spitalgasse 23 Vienna 1090 Austria; ^5^ Medical Systems Biophysics and Bioengineering Leiden Academic Centre for Drug Research Leiden University Leiden 2333 The Netherlands; ^6^ Department of Functional Materials and Catalysis Faculty of Chemistry University of Vienna Währinger Straße 42 Vienna 1090 Austria

**Keywords:** biomass carbon templates, composition, electrical properties, fungi, structure

## Abstract

Carbons form critical components in biogas purification and energy storage systems and are used to modify polymer matrices. The environmental impact of producing carbons has driven research interest in biomass‐derived carbons, although these have yield, processing, and resource competition limitations. Naturally formed fungal filaments are investigated, which are abundantly available as food‐ and biotechnology‐industry by‐products and wastes as cost‐effective and sustainable templates for carbon networks. Pyrolyzed *Agaricus bisporus* and *Pleurotus eryngii* filament networks are mesoporous and microscale with a size regime close to carbon fibers. Their BET surface areas of ≈282 m^2^ g^−1^ and ≈60 m^2^ g^−1^, respectively, greatly exceed values associated with carbon fibers and non‐activated pyrolyzed bacterial cellulose and approximately on par with values for carbon black and CNTs in addition to pyrolyzed pinewood, rice husk, corn stover or olive mill waste. They also exhibit greater specific capacitance than both non‐activated and activated pyrolyzed bacterial cellulose in addition to YP‐50F (coconut shell based) commercial carbons. The high surface area and specific capacitance of fungal carbon coupled with the potential to tune these properties through species‐ and growth‐environment‐associated differences in network and filament morphology and inclusion of inorganic material through biomineralization makes them potentially useful in creating supercapacitors.

## Graphical abstract

Fungal filaments, notably from *Agaricus bisporus* and *Pleurotus eryngii*, emerge as superior alternatives to traditional carbon sources for biogas purification and energy storage. These filaments, abundant in food and biotechnology waste, exhibit higher surface areas and specific capacitances, positioning them as effective and sustainable candidates for supercapacitor creation. This work addresses environmental and resource challenges in conventional carbon production.

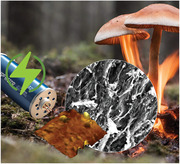

## 1 Introduction

Carbons are widely used in materials science as absorbents, e.g., for biogas purification (H_2_S removal),^[^ ^1^ ^]^ fillers for polymers^[^ ^2^ ^]^ and crucial energy storage system components.^[^ ^3^ ^]^ Typical requirements for these applications include high surface area, low density, high porosity, desired surface chemistry (for instance to ensure wettability or suitable adsorption sites), good electrical conductivity, and mechanical properties.^[^ ^4^ ^]^ High surface area carbon alternatives can also be produced by carbonization of resorcinol formaldehyde resins, aerogels or similar.^[^ ^5^ ^]^ Carbon manufacturing is varied and can utilize chemical vapor deposition, arc discharge, laser ablation, pyrolysis, activation, or graphitization processes.^[^ ^6^ ^]^

Most carbons used in materials science applications are fossil derived: carbon black, which is commonly used in tires, is typically derived from the incomplete combustion of heavy petroleum products,^[^ ^7^ ^]^ most carbon fibers used in composites are polyacrylonitrile (PAN)‐ or pitch‐based^[^ ^8^ ^]^ and graphene and carbon nanotubes (CNTs) often use fossil‐derived feedstocks.^[^ ^9^ ^]^ Consumer perception and regulation are, however, currently driving demand towards non‐fossil derived carbons.^[^ ^10^, ^11^ ^]^

The most common origins of non‐fossil‐derived carbons are ligno/cellulosic materials: wood,^[^ ^12^ ^]^ plant‐based fibers from cotton, hemp, flax or coir^[^ ^13^ ^]^ and bacterial cellulose^[^ ^14^ ^]^ can be used as templates to produce carbon networks. An example of a successful non‐fossil commercial carbon is YP‐50F (Kuraray),^[^ ^15^ ^]^ which is made from carbonized and activated coconut shell and is commonly used in supercapacitors. Research on creating carbons from biomass templates is typically directed towards either biomass with naturally organized structures, such as wood, grass and nutshell, or non‐structured raw materials, such as sucrose, pitch and plastics using hard‐/soft‐template methods, hydrothermal carbonization, chemical vapor deposition, spray pyrolysis or autogenic pressure carbonization.^[^ ^6^ ^]^ Recent studies also emphasize the use of by‐products and waste streams, such as creation of activated carbon using swine manure as a precursor^[^ ^16^, ^17^ ^]^ and porous carbons created from meat‐processing industry by‐products, such as bones, skin and scales.^[^ ^18^ ^]^ Abundant plastic waste has also aroused interest as a precursor for carbons through co‐pyrolysis with biomass, such as pinewood or sugar cane, and has the advantage of increasing surface area and improving discharge capacity in graphitic carbons.^[^ ^19^ ^]^ Additional efforts have been made to utilize biomass pyrolysis vapors, which can be achieved using a calcium citrate template.^[^ ^20^ ^]^ Key limitations of lignocellulosic material as carbon precursor are low yields (up to 30%),^[^ ^21^ ^]^ energy‐ or chemical‐intensive processing to reduce fiber sizes to the desired range^[^ ^22^ ^]^ and resource competition (e.g., food, land, or water use).^[^ ^23^ ^]^

Filamentous fungi represent a high potential yet underexplored template for producing carbon networks with key advantages over plant‐based templates. Their filamentous growth inherently ranges from micro‐ to nanoscale in size depending on the species,^[^ ^24^ ^]^ which is well suited to the production of carbon without requiring further size reduction or processing. Fungal networks often exhibit intricate and hierarchical porous structures, which could lead to carbons with unique porosities and surface areas. Species‐ and growth environment‐influenced differences in fungal filament networks could further facilitate tuning to achieve custom‐designed carbon structures.^[^ ^25^ ^]^ This is augmented by the ability of fungi to up‐concentrate and biomineralize inorganic matter,^[^ ^26^ ^]^ which has been shown to be useful for applications in energy storage systems.^[^ ^27^ ^]^

Fungal carbon templates are abundantly available as residual fungal biomass, such as spent mushroom substrate and mycelium waste^[^ ^28^ ^]^ and consequently likely much cheaper than bacterial cellulose or other plant‐based templates. Fungi‐based food and biotechnology (enzymes, biofuels, or organic acids) industry by‐products and wastes are available in volumes >170 million tons per year (5 kg of spent mushroom substrate is generated for every 1 kg of mushrooms produced)^[^ ^29^, ^30^ ^]^ that is normally downcycled into compost, animal feed or utilized for biogas production. Fungal biomass is also more rapidly producible than most plant‐based templates through industrial fermentation processes,^[^ ^31^ ^]^ which also do not compete with food production or land use. The use of fungal biomass as a template for creating carbons has, however, been almost completely neglected.

We investigated fungal biomass as a template for a carbon network. Fungal fruiting bodies served as a model for an isolated source of fungal filaments that could be carbonized to characterize this individual, high‐potential component of a spent mushroom substrate. Thermal degradation, elemental composition, morphology, physical and chemical properties of the fungal carbon network were assessed in addition to the electrical conductivity and potential for the manufacture of supercapacitor electrodes.

## 2 Results and Discussion

### 2.1 Thermal Degradation Properties of Fungal Fruiting Bodies

**Figure 1:**
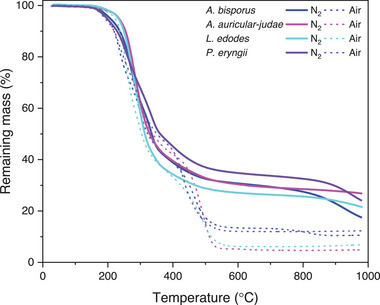
Thermograms of fungal fruiting body powders in nitrogen and air atmospheres.

*L. edodes* and *A. auricular‐judae* fungal fruiting bodies exhibited a three‐stage degradation process, while *A. bisporus* and *P. eryngii* exhibited further degradation at temperatures exceeding ≈800 °C, likely due to the partial decomposition of solid carbon oxides (Table 2) into CO_2_ and/or CO.^[^ ^32^ ^]^ All fungal materials exhibited an onset temperature of thermal degradation of ≈200–230 °C, which was in line with previously reported literature values for fungal biomass^[^ ^33^, ^34^ ^]^ (**Figure** **1**). *A. auricular‐judae* exhibited the highest char residue (27.5 wt%) and lowest inorganic content (4.9 wt%) indicating the potentially greatest fungal carbon yield. *L. edodes* also exhibited a high char residue (23.5 wt%) and low inorganic content (6.5 wt%). However, both *A. bisporus* and *P. eryngii* exhibited high inorganic contents (10.6 wt% and 12.4 wt%, respectively) and char residues of 18.2 wt% and 24.2 wt%, respectively.

### 2.2 Elemental Analysis of the Fungal Carbon

All fungal char exhibited high C (EDS: ≈43‐67 at% and etched XPS: ≈58‐70 at%) and O (EDS: ≈17‐37 at% and etched XPS: ≈18‐25 at%) content (**Tables** **1** and **2**).

**Table 1:** Energy dispersive X‐ray spectroscopy (EDS) of fungal fruiting body powders pyrolyzed using thermogravimetric analysis (TGA).

	C	O	Mg	P	Cl	K
*Agaricus bisporus*	67.2	16.8	0.3	3.3	1.0	11.6
*Auricularia auricular‐judae*	43.2	36.7	0.4	1.9	0.1	17.7
*Lentinula edodes*	64.9	19.8	0.5	3.7	0.0	11.1
*Pleurotus eryngii*	62.0	20.6	0.7	5.2	0.0	11.5

**Table 2:** Etched X‐ray photoelectron spectroscopy (XPS) of fungal fruiting body powders pyrolyzed using TGA.

	C	O	N	P	S	Cl	Si	K	Ca	Mg	Na
*Agaricus bisporus*	67.6	20.1	1.5	3.8	0.4	0.1	0.0	5.9	0.3	0.3	0.1
*Auricularia auricular‐judae*	59.2	24.8	1.8	1.6	0.2	0.2	0.9	5.5	2.1	1.5	2.3
*Lentinula edodes*	70.4	18.3	1.8	2.0	0.1	0.2	0.2	6.3	0.2	0.5	0.0
*Pleurotus eryngii*	57.8	24.8	1.7	4.2	0.8	0.2	0.0	8.8	0.5	1.3	0.1

The C content of fungal char was considerably lower than that of typical carbon‐based micro‐ and nano‐reinforcements, such as carbon fibers (typically 92–99% C),^[^ ^35^ ^]^ carbon black (typically >97% C),^[^ ^36^ ^]^ graphene (almost 100% C)^[^ ^37^ ^]^ and CNTs (typically almost 100% C).^[^ ^38^ ^]^ The lower C content of fungal chars could negatively affect their mechanical, thermal, and electrical properties and suitability as composite reinforcement or filler by disrupting the carbon lattice structure or reacting with the matrix.^[^ ^39^, ^40^ ^]^ However, the composition and surface area of the fungal chars may render them useful as absorbents/adsorbents.^[^ ^41^ ^]^

All fungal carbons also exhibited considerable potassium content (EDS: ≈11‐18 at% and etched XPS: ≈6‐9 at%) and some phosphorus (EDS: ≈1‐9 at% and etched XPS: ≈6‐9 at%). *P. eryngii* and *A. bisporus* carbons, which exhibited the highest P contents in both EDS (5.2 at% and 3.3 at%, respectively) and XPS (4.2 at% and 3.8 at%, respectively) also exhibited the highest inorganic content post pyrolisis (12.4 wt% and 10.6 wt%, respectively) while *A. auricular‐judae* and *L. edodes* carbon, which had the lowest P content in both EDS (1.9 at% and 3.7 at%, respectively) and XPS (1.6 at% and 3.0 at%, respectively), exhibited the lowest inorganic content post pyrolisis (4‐9 wt% and 6.5 wt%, respectively). Phosphorus plays an active role in fungal metabolism^[^ ^42^ ^]^ and can be incorporated into char as inorganic salts, such as potassium phosphate or potassium carbonate, during thermal decomposition.^[^ ^43^ ^]^ The inorganic fraction may consequently comprise P inorganic salts. Nitrogen was also present in all fungal carbons (etched XPS: ≈2 at%). In addition to the noted elements, *A. auricular‐judae* carbon also contained ≈2 at% Ca, Mg and Na, also likely present as inorganic salts that may have been biomineralized into the hyphae from the substrate as reported in studies investigating the production of nanopapers from mycelial biomass grown on molasses.^[^ ^26^ ^]^

The presence of inorganic salts in the fungal char did reduce the carbon content. However, P inorganic salts do have the potential to work as a flame retardant in composites^[^ ^44^ ^]^ and enhance the absorption capacity for specific contaminants or pollutants by promoting favorable interactions between the absorbent material and target substance.^[^ ^45^ ^]^ Potassium inorganic salts can also potentially promote the formation of stable carbon structures during the manufacturing process, which can also improve absorption capacity.^[^ ^46^ ^]^

### 2.3 Structural Analysis of the Fungal Carbon

**Figure 2:**
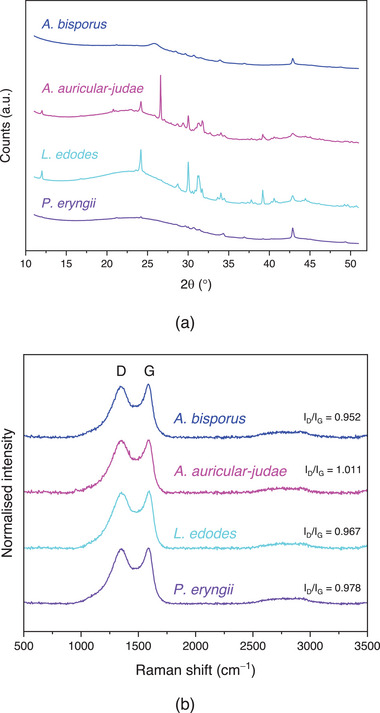
a) XRD diffractograms and b) Raman spectra of carbons derived by pyrolisis of fungal fruiting body powders.

XRD suggested that all samples comprised two types of crystalline content (**Figure** **2**a). Silica (SiO_2_) was present in all samples with quartz dust (2θ = 35°) also present on the surface. Potassium bicarbonate (KHCO_3_, 2*θ* = 23°) appeared to be the primary phase in *A. auricular‐judae* and *L. edodes* carbons. An amorphous halo was also noted in the diffractograms, associated with the presence of black carbon, which typically occurs as crystals approximately several nm in size. Subtraction of the nanocrystalline black carbon halo from the diffractogram reveals crystalline peak shapes, which can be assigned phased using Rietveld refinement (Figure S1 and Tables S1–S4, Supporting Information).

Raman spectra showed G and D bands (only associated with sp^2^ atoms) for all samples at ≈1590 and 1350 cm^−1^ in the first order phonon region (Figure 2b), which are typical for carbons. The G band indicated bond stretching of both rings and chains, and the D band breathing modes in the rings.^[^ ^47^ ^]^ The more dispersed nature of the G and D bands compared to other carbon materials, such as CNTs, indicated a more disordered carbon structure.^[^ ^48^ ^]^ A shift to higher wavenumbers and a very weak 2D band in the 2000–3000 cm^−1^ (two‐phonon processes) range also indicated a less crystalline nature or a mixture of amorphous and crystalline material.^[^ ^49^ ^]^ *A. bisporous* (0.952 ± 0.033), *L. edodes* (0.967 ± 0.022), *P. eryngii* (0.978 ± 0.032) and *A. auricular‐judae* (1.011 ± 0.067) carbons all had similar *I*_D_/*I*_G_ ratios. *I*_D_/*I*_G_ ratio can be an indicator of defect density in the material, with a higher *I*_D_/*I*_G_ ratio indicating higher defect densities.^[^ ^50^ ^]^ The similar *I*_D_/*I*_G_ ratios of the samples consequently indicated that there was no significant difference in the crystallinity and defect nature between the various fungal carbon. Their defect density was similar to pristine commercial multi‐walled CNTs,^[^ ^51^, ^52^ ^]^ slightly higher than graphene oxide, considerably higher than graphite^[^ ^53^, ^54^ ^]^ and much lower than YP‐50F (coconut shell based) commercial carbon and carbonized bacterial cellulose.^[^ ^14^ ^]^ The heterogeneous nature of the original fungal fruiting body, comprising diverse structures, such as organelles and similar, and compositions of chitin, glucans, proteins, and other polysaccharides, likely lead to uneven rates and extents of decomposition and carbonization, resulting in a higher *I*_D_/*I*_G_ ratio than carbon synthesized from more homogeneous fossil sources. Carbons dervied from fungal fruiting bodis is, however, evidently smaller and more homogenous than coconut shell or bacterial cellulose networks resulting in a more crystalline structure and thus lower *I*_D_/*I*_G_ ratio than other carbons derived from renewable resources.

### 2.4 Morphology and Physical Properties of the Fungal Carbon

**Figure 3:**
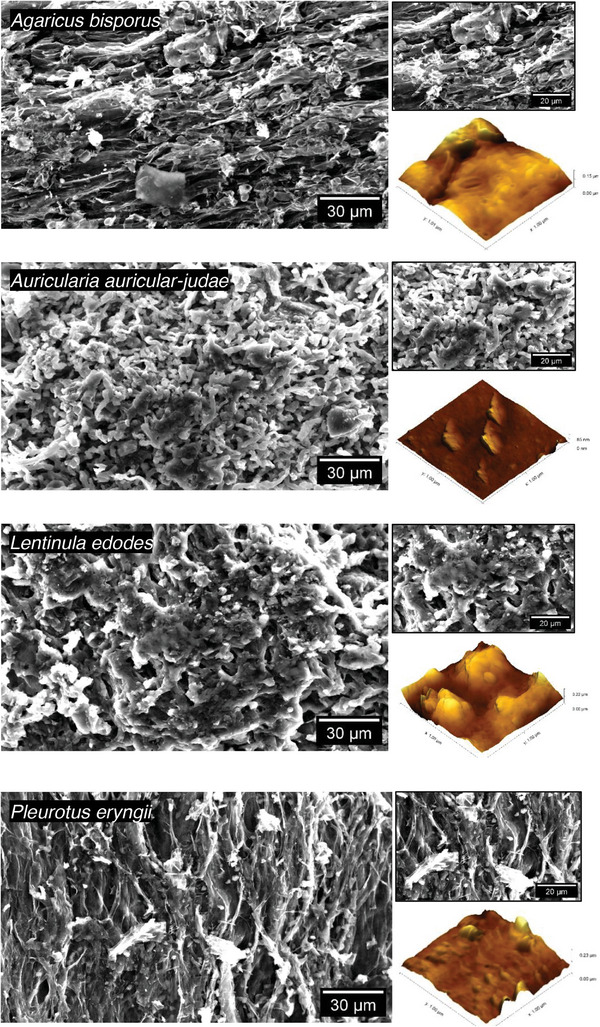
SEM micrographs and atomic force microscopy (AFM) topographies for carbon derived by pyrolisis of fungal fruiting bodies.

All carbons derived from fungal fruiting bodies retained their hyphal structures during pyrolysis to form carbonized microfilaments (**Figure** **3**). This was in line with previous investigations of the thermal degradation of mycelium that indicated negligible change in topography (SEM) and cross‐section (TEM) pre‐ and post‐pyrolysis.^[^ ^34^ ^]^ Hyphal fusion was visible in all samples, likely attributable to the collapse and aggregation of the original hyphal structures and clamp connections as the organic components decomposed and the moisture was removed.

(Individual) fungal carbon filaments had a diameter of 1–5 µm with an average of ≈2 µm for all carbon but were fused into filament aggregates and sheets. This made their size regime closer to carbon fibers (typically 5–10 µm in diameter)^[^ ^55^ ^]^ than much smaller carbon reinforcements or fillers, such as multiwalled CNTs (up to 0.1 µm),^[^ ^38^ ^]^ carbon black (0.01–0.1 µm)^[^ ^36^ ^]^ or graphene (0.00034 µm).^[^ ^37^ ^]^ These filament diameters resembled those of the hydrated hyphae in the fruiting bodies pre‐pyrolysis, which are typically 2–6 µm in *L. edodes* and *A. auricular‐judae* and 3–8 µm in *A. bisporus* and *P. eryngii*.^[^ ^42^, ^56^ ^]^

**Figure 4:**
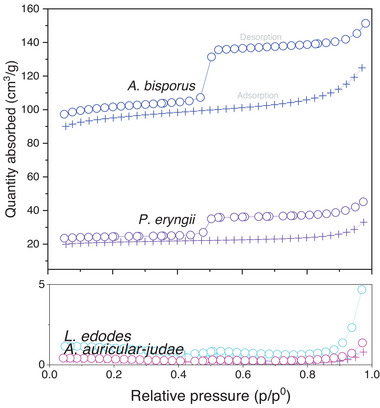
BET isotherms for fungal fruiting body powders pyrolyzed using TGA.

BET isotherms measured for all carbon indicated that the materials were mesoporous (**Figure** **4**). Notably, *A. bisporus* char had a high BET specific surface area of 282 m^2^ g^−1^. This was much higher than that associated with carbon fibers (typically 0.5–2 m^2^ g^−1^)^[^ ^55^ ^]^ and was on par with carbon black (30–300 m^2^ g^−1^)^[^ ^36^ ^]^ and some CNTs (100–1300 m^2^ g^−1^).^[^ ^38^ ^]^ It is, however, lower than common carbon‐based absorbents, such as activated carbon (800–1200 m^2^ g^−1^),^[^ ^57^ ^]^ which is typically used for water purification, gas separation or odor control, and YP‐50F commercial carbon (1692 m^2^ g^‐1^).^[^ ^15^ ^]^ That said, the material was not intentionally activated and as such the 282 m^2^ g^−1^ BET specific surface area obtained is in fact quite high and comparable with pinewood, rice husk, corn stover and olive mill waste biochars.^[^ ^58^, ^59^, ^60^ ^]^ It is also worth noting that 282 m^2^ g^‐1^ SSA is considerably higher than non‐activated carbons made from (ligno)cellulosic substrates, such as bacterial cellulose (167 m^2^ g^‐1^).^[^ ^14^ ^]^ While the SSA could be improved by activation, this leads to a massive drop in yield (10% of the original yield).^[^ ^61^ ^]^ *P. eryngii* carbon had a considerably lower BET specific surface area of 60 m^2^ g^‐1^. However, it was still much higher than *A. auricular‐judae* and *L. edodes* carbon, which exhibited no significant adsorption and hence negligible porosity. Variations in the surface area of fungal carbons are not unexpected due to the tendency for morphological features to vary greatly in size and geometry between species, as well known and documented in the literature.^[^ ^62^, ^63^ ^]^

### 2.5 Electrical Conductivity and Capacitance of the Fungal Carbon

**Figure 5:**
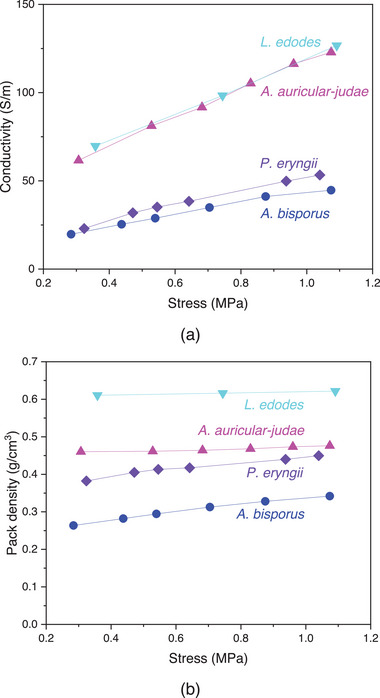
a) Electrical conductivity and b) pack density of pyrolyzed fungal fruiting body powders as a function of compressive stress.

Electrical conductivity was primarily influenced by chemical composition.^[^ ^64^ ^]^ *L. edodes* and *A. auricular‐judae* carbons exhibited similar electrical conductivities that were higher than those associated with the other carbonized fungal biomass (**Figure** **5**a). All fungal carbons exhibited comparable conductivity to carbon black and graphene.^[^ ^65^ ^]^ Electrical conductivity was also dependent on pack density (Figure 5b). Carbon derived from *A. auricular‐judae* exhibited higher intrinsic or specific conductivity (normalized to pack density) than that derived from *L. edodes*. Fungal carbon powders would need to be ground and sieved in order for a comparison with commercial carbon produced with controlled manufacturing and quality control processes to be fair.

**Figure 6:**
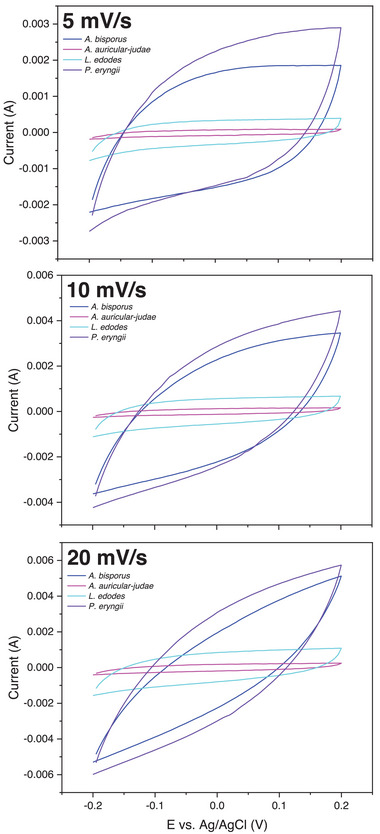
Cyclic voltammetry profiles for pyrolyzed fungal fruiting body powders at scan rates of a) 5 mV s^−1^, b) 10 mV s^−1^ and c) 20 mV s^−1^.

The CV profiles of all fungal carbons indicated a reversible process without side reactions, such as oxidation or reduction, during the cycles (**Figure** **6**). *A. auricular‐judae* and *L. edodes* carbons exhibited CV profiles indicating electrostatic attraction‐based electrode capacitive resistance,^[^ ^66^ ^]^ while the CV profiles of *A. bisporus* and *P. eryngii* carbons indicated intrinsic electrical resistance. Despite their lower electrical conductivity, *A. bisporus* and *P. eryngii* carbons exhibited much greater specific capacitance than the other carbons, especially at low scan rates (**Table** **3**), which could be due to the higher BET surface area of these carbons (282 and 60 m^2^ g^‐1^, respectively) and electrical conductivity. That said, *L. edodes* carbon exhibited a much higher specific capacitance than *A. auricular‐judae* carbon despite having similarly negligible BET surface area. A higher metal ion content in *L. edodes* carbon could explain this discrepancy. Higher specific capacitance carbons (*A. bisporus* and *P. eryngii*) were also more sensitive to differences associated with scan rate, likely due to the porous nature of these carbon samples whereby the current predominantly flows at the external surface of these materials at higher scan rates.

**Table 3:** Specific capacitance (F g^‐1^) of pyrolyzed fungal fruiting body powders at 5, 10 and 20 mV s^‐1^ scan rates compared to non‐activated and activated bacterial cellulose and commercial YP‐50F carbon.

Sample	Specific capacitance [F g^‐1^]		
	5 mV s^‐1^	10 mV s^‐1^	20 mV s^‐1^
YP‐50F^a)^	53 ± 1	50 ± 1	45 ± 1
Pyrolyzed bacterial cellulose (non‐activated)^a)^	15 ± 1	11 ± 1	6 ± 1
Pyrolyzed bacterial cellulose (activated)^a)^	32 ± 1	28 ± 1	23 ± 1
*Agaricus bisporus carbon*	41 ± 7	26 ± 9	12 ± 4
*Auricularia auricular‐judae carbon*	7 ± 4	7 ± 3	6 ± 1
*Lentinula edodes carbon*	22 ± 2	18 ± 1	12 ± 2
*Pleurotus eryngii carbon*	43 ± 1	34 ± 5	23 ± 4

^a)^ Literature values from Lee et al.^[^ ^14^ ^]^

The specific capacitance of *P. eryngii* and *A. bisporus* carbons can be considered moderate for supercapacitor applications. They considerably exceed values obtained for non‐activated bacterial cellulose derived carbon and even exceed those associated with activated pyrolyzed bacterial cellulose.^[^ ^14^ ^]^ They are also only slightly lower than the capacitance of commercial YP‐50F carbon.^[^ ^14^ ^]^ Their specific capacitances are, however, lower than traditional activated carbons which exhibit specific capacitance of 50–150 F g^‐1^. Capacitance can likely be increased (to >300 F g^‐1^) by biomineralizing the fungal filaments through growth in a mineral‐rich growth medium prior to pyrolysis.^[^ ^27^ ^]^

## 3 Conclusion

*Agaricus bisporus* and *Pleurotus eryngii* fungal biomass could be utilized as templates for carbon networks with yields of ≈18 and ≈24 wt% including an inorganic content of ≈10 and ≈12 wt%, respectively, following pyrolysis. Carbonized fungal biomass exhibited networks of microfilaments resembling the pre‐pyrolysis mycelium network and including evidence of hyphal fusion, providing them with a size regime close to carbon fibers. These carbon networks were mesoporous with BET surface areas of ≈282 m^2^ g^‐1^ for *A. bisporus* and ≈60 m^2^ g^‐1^ for *P. eryngii* carbons, which was considerably greater than values associated with carbon fibers and non‐activated pyrolyzed bacterial cellulose and approximately on par with values for carbon black and CNTs in addition to pyrolyzed pinewood, rice husk, corn stover and olive mill waste. Notably, both *A. bisporus* and *P. eryngii* derived carbons exhibited greater specific capacitance than both non‐activated and activated pyrolyzed bacterial cellulose in addition to YP‐50F (coconut shell based) commercial carbons. Conversely, *Lentinula edodes* and *Auricularia auricular‐judae* carbons exhibited low specific capacitance due to their low surface area but higher electrical conductivity than *A. bisporus*‐ and *P. eryngii*‐based carbons. The high surface area and specific capacitance of *A. bisporus* and *P. eryngii* carbonized fungal biomass coupled with the abundance of mycelium‐based by‐products and wastes from the food and biotechnology sectors, could be useful in creating supercapacitors in a much more cost‐effective manner than would be possible with bacterial cellulose or similar. Notable abilities of fungi, such as radically different network and filament morphologies based on species and growth conditions and their ability to biomineralize metals, salts and other inorganic material could also facilitate fungal carbons with tunable morphologies, surface areas and electrical properties.

## 4 Experimental Section

### Materials

*Agaricus bisporus* (white button, Pielachtaler Pilze, Austria) and *Pleurotus eryngii* (king oyster, Marchfelder Bio Edelpilze GmbH, Austria) mushrooms were purchased in a hydrated state from a local supermarket. *Auricularia auricular‐judae* (wood ear, Kreyenhop & Kluge GmbH & Co. KG, Germany) and *Lentinula edodes* (shiitake, Asia Express Food, Netherlands) mushrooms were purchased in a dehydrated state from a local Asian specialty food store. Acetylene black (50% compressed, purity 99.9%,) was purchased from Alfa Aesar (Ward Hill, USA), poly(vinylidene fluoride) (PVDF, Kynar 711) was kindly provided by Akerma (Colombes, France), 1‐methyl‐2‐pyrrolidone (NMP) was purchased from Sigma Aldrich (St. Louis, USA) and nickel foam sheet (Ni‐4753 Nickel foam sheet) from Recemat BV (Dodewaard, The Netherlands) for creating supercapacitors.

### Pyrolysis of Fungal Fruiting Bodies to Form Fungal Char

*A. bisporus* and *P. eryngii* mushrooms were first washed with water and then allowed to air dry for 7 d. All dehydrated mushrooms were then ground into a fine powder using a coffee grinder.

Fungal fruiting body thermal degradation properties were assessed using a TA Instruments TGA550 thermogravimetric analyzer (TGA). Dry mushroom powder samples of ≈10 mg were placed in an alumina high temperature crucible and heated from 30 to 1000 °C at a heating rate of 20 K min^‐1^. Samples were tested in air and nitrogen atmospheres (both 25 mL min^−1^). Thermal degradation properties were then analyzed using TA Universal Analysis (v. 4.5A b. 4.5.0.5).

Larger quantities of pyrolyzed mushroom powder for additional tests were obtained using a Mettler Toledo TGA/DSC 1 STAR^e^ system and deep alumina crucibles (maximum sample mass of 4 g). Samples were run isothermally at 1000 °C for 15 min in a nitrogen atmosphere.

### Morphological and Elemental Analysis of the Fungal Carbon

Scanning electron microscopy (SEM) imaging and energy dispersive spectroscopy (EDS) elemental analysis of each sample was performed using a Philips XL30 scanning electron microscope with an EDAX energy dispersive X‐ray spectrometer attached. An accelerating voltage of 30 kV was used. The EDS spectra were analyzed using EDAX Genisis software. An average spectrum was obtained based on individual spectra from 12 different sites. One hundred individual filaments (not filament aggregates) were measured from SEM micrographs using the line and measure function in the Fiji distribution of ImageJ (v. 1.53t) across three sites (micrographs) for each sample and the results averaged and standard deviation calculated.

Prior to atomic force microscopy (AFM), pyrolyzed powder samples were suspended in isopropyl alcohol and an ultrasonic bath used to separate clusters of particles in the solution. 1 mL of the suspension was then deposited on a piece of silicon using a custom‐built spin coater and the prepared sample applied to carbon tape. AFM was performed using a FusionScope (Quantum Design, San Diego, USA), AFM/SEM combined instrument. The AFM tip was positioned on top of the structure using SEM and topography recorded using amplitude‐modulation at a scan speed of 0.5 Hz. AFM was performed under high vacuum using silicon tip self‐sensing cantilevers with piezo‐resistive readout (AMG Technology Ltd., Botevgrad, Bulgaria). The resonance frequency of the cantilever ranged between 450 and 650 kHz and the stiffness between 15 and 150 N m^‐1^. AFM data were processed using Gwyddion (v. 2.59) with plane subtraction, alignment of rows, correction of horizontal scars and removal of polynomial background applied.

X‐ray photoelectron spectroscopy (Nexsa, Thermofisher) was performed to investigate the surface composition of pyroysed powder samples using Al Kα radiation at 72 W. Thirty passes were made using pass energies of 200 eV for survey spectra and 50 eV for high‐resolution spectra, respectively, and a spot size of 400 µm. “Standard Lens Mode” and CAE Analyzer Mode were used with the integrated flood gun. Surfaces were etched with argon clusters (1000 atoms) for 60 s at 6000 eV.

Nitrogen adsorption/desorption isotherms of the pyrolyzed powder samples were measured at −196 °C using a TriStar II 3020 (Micrometrics Ltd., Aachen, DE). Samples were degassed (Flow prep 06, Micromeritics, Aachen, DE) at 120 °C for 16 h in continuous dry N_2_ flow prior to the measurement to remove adsorbed water molecules.

### Structural Analysis of the Fungal Carbon

X‐ray diffraction was performed on an Empyrean powder diffractometer (PANalytical, Almelo, The Netherlands) in Theta/Theta mode on a reflection‐transmission spinner in reflection mode, using a Bragg‐Brentano mirror. The anode material was Cu and measuring temperature ≈22 °C. All measurements were continuous scans from 11 to 51° (two theta), with a step size of 0.0131°, count time per step of 10 times 549 s and spinning rotation time of 8 s. Search and match analysis was completed following subtraction of the amorphous component, which was identified as nano‐crystalline black carbon (described in the literature^[^ ^67^ ^]^). Rietveld refinement was then performed for the corresponding phases.

Raman spectroscopy was performed with a WITec alpha 300A micro‐Raman device and a 100×/0.9 objective. Measurements were performed in the backscattered geometry employing a frequency doubled Nd:YAG laser at a wavelength of 532 nm, an average power of 2 mW, and an acquisition time of 100 s. Powders were assessed as‐received over five replicate sites per sample. Spectra were corrected by removal of cosmic rays and background, normalized to an area of 1, and averaged.

### Electrical Conductivity and Capacitance of the Fungal Carbon

Through‐thickness conductivity was measured as described by Marinho, Ghislandi.^[^ ^65^ ^]^ A poly(methyl methacrylate) (PMMA) ring with a thickness of 1 cm and inner diameter of 1.5 cm was fixed to a copper plate. Approximately 0.05 g of ground *A. bisporus* and *P. eryngii* and 0.27 g of *L. edodes* and *A. auricular‐judae* (too stiff to be compacted) pyrolized fungal fruiting body powder, respectively, was inserted into the PMMA ring and a copper plunger with a height of 2 cm and diameter of 1.5 cm used to compact the powder from 50 to 180 N within the compression jig of a universal mechanical testing frame (Instron 5569, Instron, Germany) equipped with a 1 kN load cell. Pack thickness of the powder disk was assessed using the mechanical tester. Powder resistance (*R*) was measured using 4‐electrode mode on a source measure unit (Keithley 2450, Keithley, Austria) and powder conductivity (κ) calculated based on the current *I*, powder contact area (*A* = 176.7 mm^2^) and *R* (Equation 1). Pack density was calculated as powder mass divided by volume. Pack density and powder conductivity were plotted as *f*(σ), where σ is force divided by contact area.

Cyclic voltammetry (CV) was performed adapting a method reported by Leeet al.^[^ ^14^ ^]^ Acetylene black and PVDF with a weight ratio of 87:10:3 were mixed with 1‐methyl‐2‐pyrrolidone and the suspension sonicated in an ultrasound bath for 5 min. The suspension was then applied to a piece of Ni foam and the solvent dried at room temperature for 24 h and then at 80 °C for 2 h. CV was completed using a Gamry Reference 600 potentiostat (Munich, Germany). The three‐electrode cell comprised the coated Ni foam (working electrode), Pt wire (counter electrode) and Ag/AgCl (reference electrode). The CV profile was obtained between −0.2 and 0.2 V at scan rates of 5, 10 and 20 mV s^‐1^. Specific capacitance, *C*, was calculated based on the anodic (*I*_a_) and cathodic (*I*_c_) current, material mass (*m*) and scan rate (d*E*/d*t*) (Equation 2).

(1) $$\kappa \;=\frac{I}{A\cdot R}\;$$

(2) $$C\;=\frac{I_{a}-I_{c}}{2\times m\times \frac{dE}{dt}}$$

## Conflict of Interest

The authors declare no conflict of interest.

## Supporting information

Supporting Information

## Data Availability

The data that support the findings of this study are available from the corresponding author upon reasonable request.
