# Supplementary material for: Fungal Carbon: A Cost‐Effective Tunable Network Template for Creating Supercapacitors
Source: Glob Chall. 2024 Mar 18;8(4):2300315. doi: 10.1002/gch2.202300315 (PMC11009424; doi:10.1002/gch2.202300315)
Supplement: Supplementary file 1 — Supporting Information [file GCH2-8-2300315-s001.pdf]

Global Challenges

Open Access

Supporting Information

for *Global Challenges.*, DOI 10.1002/gch2.202300315

Fungal Carbon: A Cost-Effective Tunable Network Template for Creating Supercapacitors

Mitchell P. Jones, Qixiang Jiang, Andreas Mautner, Aida Naghilou, Alexander Prado-Roller, Marion Wolff, Thomas Koch, Vasiliki-Maria Archodoulaki and Alexander Bismarck**

Electronic supplementary material for:

Fungal carbon: A cost-effective tuneable network template for creating supercapacitors

Mitchell P. Jones[1,*], Qixiang Jiang[2], Andreas Mautner[2,3], Aida Naghilou[4], Alexander Prado-Roller[5], Marion Wolff[1], Thomas Koch[1], Vasiliki-Maria Archodoulaki[1], Alexander Bismarck[2,*]

[1] Institute of Materials Science and Technology, Faculty of Mechanical and Industrial Engineering, TU Wien, Gumpendorferstrasse 7, Objekt 8, 1060 Vienna, Austria
[2] Polymer & Composite Engineering (PaCE) Group, Institute of Materials Chemistry and Research, Faculty of Chemistry, University of Vienna, Währinger Straße 42, 1090, Vienna, Austria
[3] Institute for Environmental Biotechnology, Department IFA, University of Natural Resources and Life Sciences Vienna, Konrad-Lorenz-Straße 20, 3430 Tulln an der Donau, Austria
[4] Department of Plastic, Reconstructive and Aesthetic Surgery, Medical University of Vienna, Spitalgasse 23, 1090 Vienna, Austria
[5] Department of Functional Materials and Catalysis, Faculty of Chemistry, University of Vienna, Währinger Straße 42, 1090 Vienna, Austria
* Correspondence: mitchell.jones@tuwien.ac.at (M.P. Jones), alexander.bismarck@univie.ac.at (A. Bismarck)

List of Figures

Figure S1. Phases with Rietveld refinement: (a) *Agaricus bisporus*, one phase refined (ICSD 98-007-0007); (b) *Auricularia auricula-judae*, two phases refined (ICSD 98-008-0552 and 98-007-0007); (c) *Lentinula edodes*, two phases refined (ICSD 98-008-0552 and 98-007-0007); and (d) *Pleurotus eryngii*, one phase refined (ICSD 98-007-0007). Strongest reflections are labelled with hkl. Several peaks could not be identified.

List of Tables

Table S1. *Agaricus bisporus* peak list

Table S2. *Auricularia auricula-judae* peak list. Peaks of 98-007-0007 (Quarz, low) are bolded

Table S3. *Lentinula edodes* peak list. Peaks of 98-007-0007 (Quarz, low) are bolded

Table S4. *Pleurotus eryngii* peak list

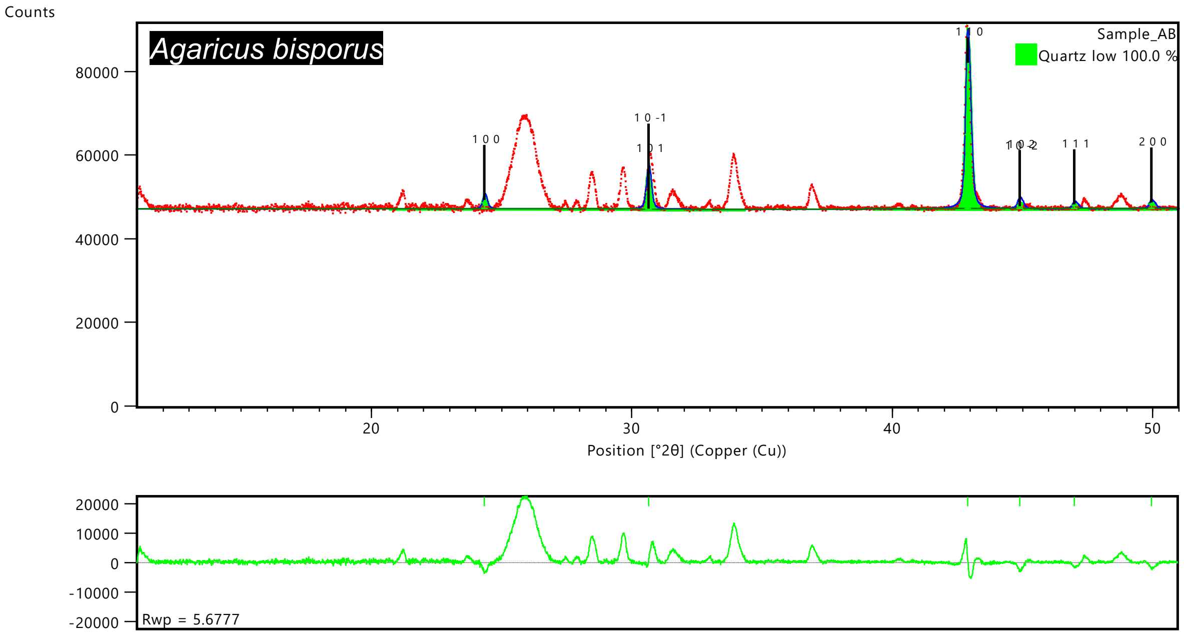
Counts
Agaricus bisporus
Sample_AB
Quartz low 100.0 %
80000
60000
40000
20000
0
20
30
40
50
Position [°2θ] (Copper (Cu))
20000
10000
0
-10000
-20000
Rwp = 5.6777

(a)

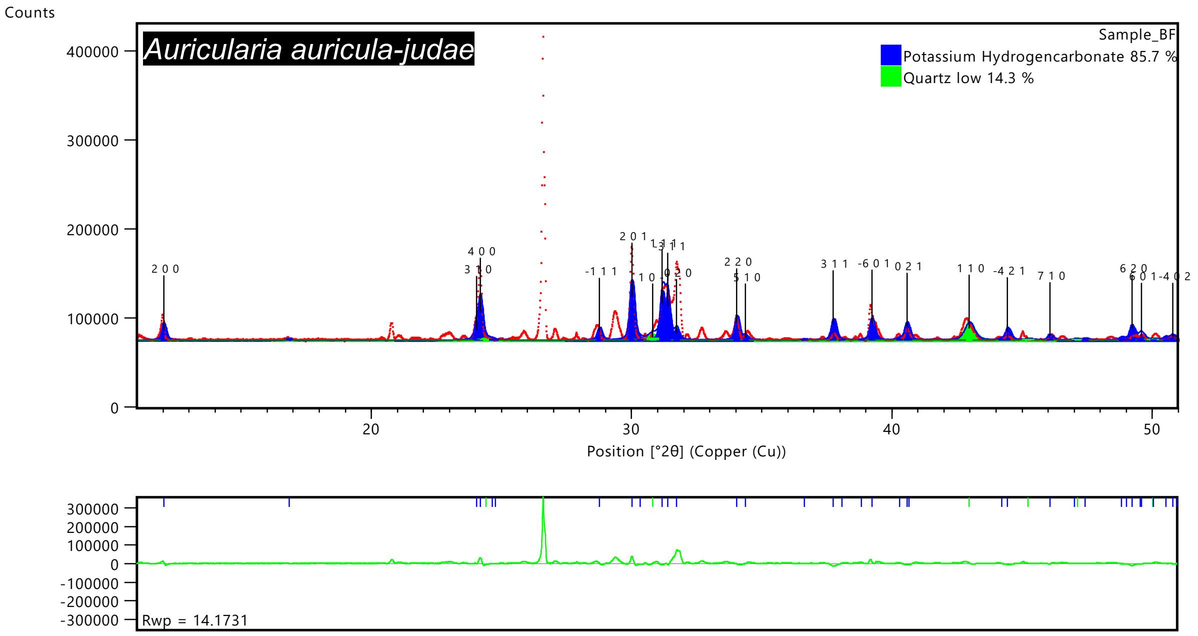
Counts
Auricularia auricula-judae
Sample_BF
Potassium Hydrogencarbonate 85.7 %
Quartz low 14.3 %
400000
300000
200000
100000
0
20
30
40
50
Position [°2θ] (Copper (Cu))
300000
200000
100000
0
-100000
-200000
-300000
Rwp = 14.1731

(b)

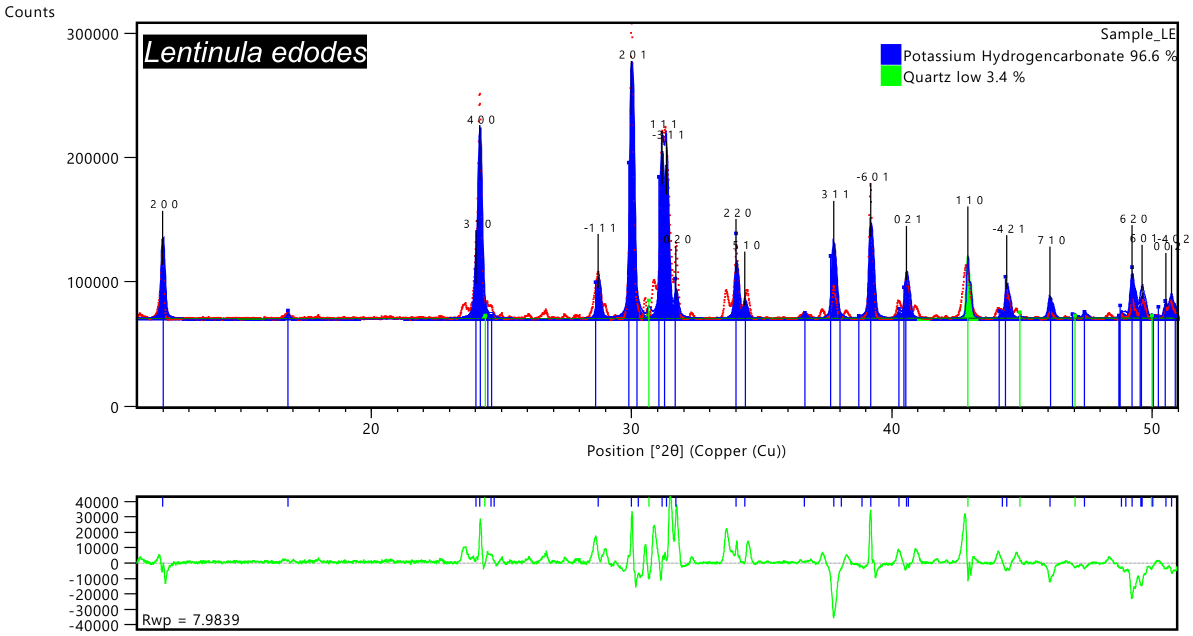

(c)

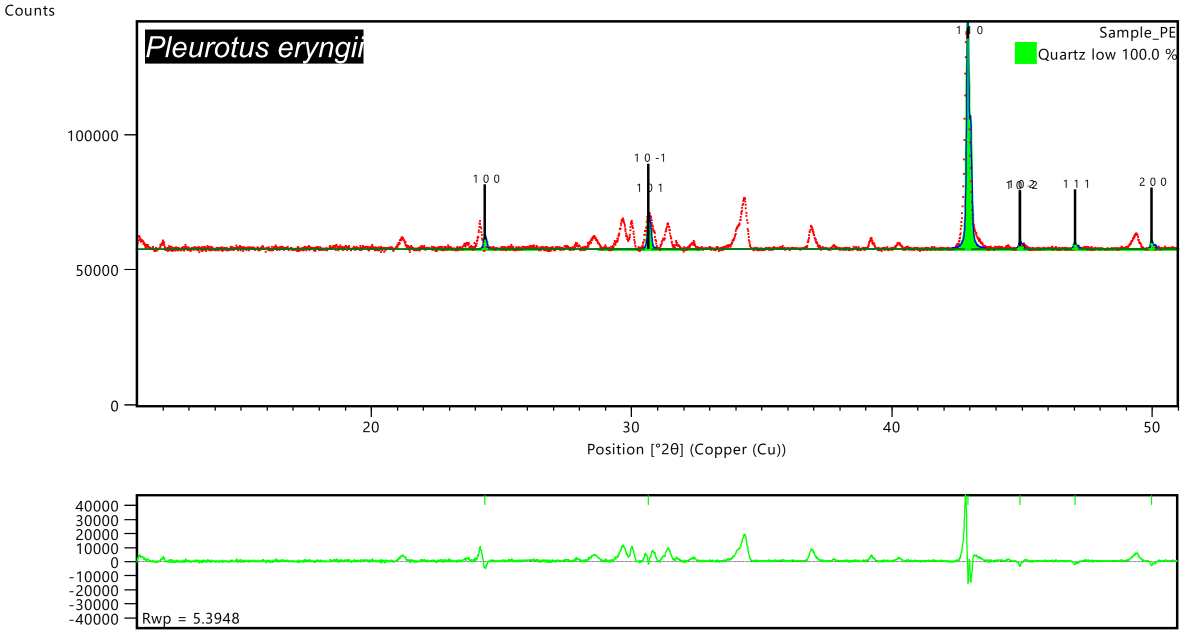

(d)

Figure S1. Phases with Rietveld refinement: (a) *Agaricus bisporus*, one phase refined (ICSD 98-007-0007); (b) *Auricularia auricula-judae*, two phases refined (ICSD 98-008-0552 and 98-007-0007); (c) *Lentinula edodes*, two phases refined (ICSD 98-008-0552 and 98-007-0007); and (d) *Pleurotus eryngii*, one phase refined (ICSD 98-007-0007). Strongest reflections are labelled with hkl. Several peaks could not be identified.

Table S1. *Agaricus bisporus* peak list

No.	Pos. [°2θ]	Height [cts]	Area [cts*°2θ]	h	k	l
1	24.3427	2567.75	627.82	1	0	0
2	30.6304	7719.83	1887.53	1	0	-1
3	30.6304	479.22	117.17	1	0	1
4	42.8848	35409.06	8657.64	1	1	0
5	44.8873	1025.37	250.71	1	0	-2
6	44.8873	1362.56	333.15	1	0	2
7	46.996	1479.4	361.72	1	1	1
8	49.9479	1890.33	462.19	2	0	0

Table S2. *Auricularia auricula-judae* peak list. Peaks of 98-007-0007 (Quarz, low) are bolded

No.	Pos. [°2θ]	Height [cts]	Area [cts*°2θ]	h	k	l
1	12.034	12966.02	3241.17	2	0	0
2	16.8371	1676.72	419.14	1	1	0
3	24.0415	12365.13	3090.97	3	1	0
4	24.1894	33031.33	8256.99	4	0	0
**5**	**24.4148**	**1415.33**	**775.8**	**1**	**0**	**0**
6	24.6447	603.16	150.77	0	0	1
7	24.7673	169.35	42.33	-2	0	1
8	28.7636	10289.02	2571.99	-1	1	1
9	30.0108	49973.89	12492.2	2	0	1
10	30.3166	1005.92	251.45	-4	0	1
**11**	**30.7965**	**4390.51**	**2406.61**	**1**	**0**	**-1**
**12**	**30.7965**	**272.59**	**149.42**	**1**	**0**	**1**
13	31.1771	42026.8	10505.63	1	1	1
14	31.3744	38420.09	9604.04	-3	1	1
15	31.7224	9423.92	2355.74	0	2	0
16	34.0375	21051.31	5262.29	2	2	0
17	34.3601	4252.53	1063.02	5	1	0
18	36.6287	1102.72	275.65	6	0	0
19	37.7525	18761.46	4689.89	3	1	1
20	38.0859	183.88	45.97	-5	1	1
21	38.8227	255.82	63.95	4	0	1
22	39.2295	19903.79	4975.44	-6	0	1
23	40.282	2141.6	535.35	4	2	0
24	40.5722	15938.08	3984.11	0	2	1
25	40.6509	683.89	170.95	-2	2	1
**26**	**42.957**	**14167.33**	**7765.65**	**1**	**1**	**0**
27	44.2241	1572.22	393.01	2	2	1
28	44.444	11362.12	2840.24	-4	2	1
**29**	**45.2302**	**584.21**	**320.23**	**1**	**0**	**-2**
**30**	**45.2302**	**781.56**	**428.4**	**1**	**0**	**2**
31	46.0594	5289.47	1322.23	7	1	0
32	47.0079	706	176.48	5	1	1

**33**	**47.1335**	**788.04**	**431.96**	**1**	**1**	**1**
34	47.4266	1453.08	363.23	-7	1	1
35	48.8155	2706.7	676.61	1	3	0
36	49.0043	417.53	104.37	-2	0	2
37	49.2157	14162.12	3540.17	6	2	0
38	49.5341	2786.45	696.54	8	0	0
39	49.5794	5094.94	1273.6	6	0	1
**40**	**50.0199**	**1044.94**	**572.77**	**2**	**0**	**0**
41	50.0487	161.64	40.4	-8	0	1
42	50.5162	3245.92	811.4	0	0	2
43	50.7811	5065.94	1266.36	-4	0	2
44	50.9656	154.02	38.5	4	2	1

Table S3. *Lentinula edodes* peak list. Peaks of 98-007-0007 (Quarz, low) are bolded

No.	Pos. [°2θ]	Height [cts]	Area [cts*°2θ]	h	k	l
1	11.9864	44563.76	8249.06	2	0	0
2	16.7934	3022.14	559.42	1	1	0
3	24.0104	28781.64	5327.68	3	1	0
4	24.1611	112802.3	20880.49	4	0	0
**5**	**24.3606**	**1938.77**	**255.86**	**1**	**0**	**0**
6	24.6062	1963.34	363.43	0	0	1
7	24.7113	552.69	102.31	-2	0	1
8	28.7236	25791.94	4774.27	-1	1	1
9	29.9979	164937.1	30531.01	2	0	1
10	30.2601	3334.7	617.28	-4	0	1
**11**	**30.6515**	**5506.01**	**726.63**	**1**	**0**	**-1**
**12**	**30.6515**	**341.8**	**45.11**	**1**	**0**	**1**
13	31.1569	109213.9	20216.24	1	1	1
14	31.3261	100357.2	18576.82	-3	1	1
15	31.6991	15961.71	2954.62	0	2	0
16	34.0188	37764.77	6990.52	2	2	0
17	34.3473	11713.95	2168.33	5	1	0
18	36.6218	3758.47	695.72	6	0	0
19	37.7567	52457.37	9710.22	3	1	1
20	38.0425	517.01	95.7	-5	1	1
21	38.8362	852.33	157.77	4	0	1
22	39.1849	66687.4	12344.3	-6	0	1
23	40.2755	4364.77	807.95	4	2	0
24	40.5592	32468.86	6010.21	0	2	1
25	40.6267	1394.21	258.08	-2	2	1
**26**	**42.9141**	**48128.59**	**6351.54**	**1**	**1**	**0**
27	44.2296	3392.05	627.89	2	2	1
28	44.4181	24612.17	4555.88	-4	2	1
**29**	**44.9163**	**718.38**	**94.81**	**1**	**0**	**-2**
**30**	**44.9163**	**955.21**	**126.06**	**1**	**0**	**2**

31	46.0688	15833.21	2930.84	7	1	0
**32**	**47.0276**	**1154.31**	**152.33**	**1**	**1**	**1**
33	47.0383	2098.92	388.52	5	1	1
34	47.3972	4343.07	803.93	-7	1	1
35	48.8195	4603.37	852.12	1	3	0
36	48.977	1353.82	250.6	-2	0	2
37	49.2273	32783.52	6068.46	6	2	0
38	49.5518	9464.85	1752.01	8	0	0
39	49.6204	17074.47	3160.6	6	0	1
**40**	**49.9819**	**1426.98**	**188.32**	**2**	**0**	**0**
41	50.0227	544.47	100.78	-8	0	1
42	50.5122	10539.02	1950.85	0	0	2
43	50.7394	16494.64	3053.27	-4	0	2
44	50.9942	360.38	66.71	4	2	1

Table S4. *Pleurotus eryngii* peak list

No.	Pos. [°2θ]	Height [cts]	Area [cts*°2θ]	h	k	l
1	24.3575	4128.69	544.86	1	0	0
2	30.6484	11949.93	1577.03	1	0	-1
3	30.6484	741.83	97.9	1	0	1
4	42.9111	81629.98	10772.73	1	1	0
5	44.9133	1567.87	206.91	1	0	-2
6	44.9133	2084.74	275.12	1	0	2
7	47.0247	2429.58	320.63	1	1	1
8	49.979	3038.4	400.98	2	0	0
